# Development and validation of a nomogram for predicting hospitalization longer than 14 days in pediatric patients with ventricular septal defect—a study based on the PIC database

**DOI:** 10.3389/fphys.2023.1182719

**Published:** 2023-07-04

**Authors:** Jia-Liang Zhu, Xiao-Mei Xu, Hai-Yan Yin, Jian-Rui Wei, Jun Lyu

**Affiliations:** ^1^ Department of Intensive Care Unit, The First Affiliated Hospital of Jinan University, Guangzhou, China; ^2^ Guangzhou Women and Children’s Medical Center, Guangzhou Medical University, Guangzhou, China; ^3^ Department of Clinical Research, The First Affiliated Hospital of Jinan University, Guangzhou, China; ^4^ Guangdong Provincial Key Laboratory of Traditional Chinese Medicine Informatization, Guangzhou, China

**Keywords:** ventricular septal defect, nomogram, PIC database, retrospective study, XGBoost

## Abstract

**Background:** Ventricular septal defect is a common congenital heart disease. As the disease progresses, the likelihood of lung infection and heart failure increases, leading to prolonged hospital stays and an increased likelihood of complications such as nosocomial infections. We aimed to develop a nomogram for predicting hospital stays over 14 days in pediatric patients with ventricular septal defect and to evaluate the predictive power of the nomogram. We hope that nomogram can provide clinicians with more information to identify high-risk groups as soon as possible and give early treatment to reduce hospital stay and complications.

**Methods:** The population of this study was pediatric patients with ventricular septal defect, and data were obtained from the Pediatric Intensive Care Database. The resulting event was a hospital stay longer than 14 days. Variables with a variance inflation factor (VIF) greater than 5 were excluded. Variables were selected using the least absolute shrinkage and selection operator (Lasso), and the selected variables were incorporated into logistic regression to construct a nomogram. The performance of the nomogram was assessed by using the area under the receiver operating characteristic curve (AUC), Decision Curve Analysis (DCA) and calibration curve. Finally, the importance of variables in the model is calculated based on the XGboost method.

**Results:** A total of 705 patients with ventricular septal defect were included in the study. After screening with VIF and Lasso, the variables finally included in the statistical analysis include: Brain Natriuretic Peptide, bicarbonate, fibrinogen, urea, alanine aminotransferase, blood oxygen saturation, systolic blood pressure, respiratory rate, heart rate. The AUC values of nomogram in the training cohort and validation cohort were 0.812 and 0.736, respectively. The results of the calibration curve and DCA also indicated that the nomogram had good performance and good clinical application value.

**Conclusion:** The nomogram established by BNP, bicarbonate, fibrinogen, urea, alanine aminotransferase, blood oxygen saturation, systolic blood pressure, respiratory rate, heart rate has good predictive performance and clinical applicability. The nomogram can effectively identify specific populations at risk for adverse outcomes.

## Introduction

Ventricular septal defect (VSD) is the most common form of congenital heart disease, accounting for approximately 40% of congenital heart disease cases (Penny and Vick, 2011). VSD leads to blood shunting, resulting in increased pulmonary blood circulation volume and pathological changes in the pulmonary blood vessels, which makes children with VSD particularly prone to pulmonary infection. As the disease progresses, when the pulmonary circulation pressure is higher than the systemic circulation pressure, the blood flows from the right to the left ventricle, which increases the preload of the left ventricle that easily leads to heart failure. The alveolar development of young infants is not perfect, the respiratory system is immature, and the synthesis function of alveolar type II epithelial cells is deficient, resulting in less alveolar surfactant production, and so the respiratory function is immature. Both pulmonary hypoplasia and infection may lead to a decrease in the arterial partial pressure of oxygen, which further leads to respiratory rate changes.

Occurrence of infection may be a risk factor for prolonged hospital stay in patients with VSD. Extended hospital stays may further increase the likelihood of nosocomial infections. Pulmonary infection may cause pulmonary interstitial edema, resulting in decreased pulmonary ventilation and hypoxemia, which ultimately leads to rapid breathing. The infection diagnoses of some cases are not clear, making it difficult to determine whether a hospital stay was prolonged. However, the respiratory rate (Rr) is an easy metric to monitor and its measurement accuracy is high, and hence it may have predictive value for the length of hospital stay. A study found that a higher respiratory rate on admission was associated with an increased risk of in-hospital mortality in patients admitted from nursing homes (Myint et al., 2011). We therefore set the outcome of the present study as a hospital stay longer than 14 days. We aimed to develop a nomogram to assess the risk of hospitalization for longer than 14 days in pediatric patients with VSD. We hope that clinicians can make timely adjustments to treatment regimens based on changes in risk factors in the nomogram model to reduce hospital stays in pediatric patients with VSD.

## Methods

### Data source

This is a single center retrospective study. Data for the study were extracted from the Paediatric Intensive Care (PIC) database. This is a large, single-center, pediatric-specific database that includes the clinical data of all patients admitted to different ICUs at the Children’s Hospital Zhejiang University School of Medicine during 2010–2018, and includes the admission information of 13,499 cases from among 12,881 different pediatric patients (Yang et al., 2020). The data include patient demographics, drug use, fluid balance, comprehensive laboratory test results, and microbiological information obtained throughout a hospital stay. It also includes vital sign information collected from the anesthesia information management system during surgery. According to the requirements of the database, we completed the relevant training courses and passed the examination to obtain an access certificate (no. 45848364). The private information of patients has been altered in the PIC database, and so this study did not require approval from the hospital ethics committee.

### Participant selection

All of the included patients had been diagnosed with a ventricular septal defect.

### Study metrics

We used Structured Query Language to extract metrics within 24 h of admission from the PIC database. These included the basic information, vital signs, and laboratory test indicators of the patients. Basic information included sex, marital status, language. Since the subjects of this study were infants and young children, we did not include the language and marital statuses. Vital-sign information included body temperature (T), heart rate (Hr), respiratory rate, systolic blood pressure (SBP), diastolic blood pressure (DBP), and blood oxygen saturation (SpO2). We also collected laboratory variables including adenosine deaminase (ADA), serum albumin, alanine aminotransferase (ALT), serum creatinine, total bilirubin (Tbil), globulin, hemoglobin, lymphocyte count, red blood cell count (RBC), white blood cell count (WBC), platelet count (PLT), eosinophils, D-dimer, fibrinogen, international normalized ratio (INR), serum sodium (Na), brain natriuretic peptide (BNP) and bicarbonate (Wu et al., 2021).

### Research design

We used variance inflation factor (VIF) to assess whether there was collinearity between variables, and excluded variables with VIF values > 5. The existence of a variable with multicollinearity means that the corresponding information provided by this variable is redundant in the presence of other variables. The variables were incorporated into the least absolute shrinkage and selection operator model (Lasso). The LASSO regression method was used to reduce the dimensionality of variables with certain correlations, and variables with non-zero coefficient characteristics were selected. Calculate the cut-off point of continuous variables, and divide the continuous variables into two groups according to the cut-off point, one is above the cut-off point, and the other is below the cut-off value. The Odds ratio of the variables was calculated using a logistic regression model and represented by a forest plot. Eligible variables were incorporated into logistic regression to build the nomogram. The performance of the nomogram was assessed using the area under the receiver operating characteristic curve (AUC), with AUC >0.7 indicating good model performance. Calibration curves and Decision Curve Analysis (DCA) were used to evaluate the predictive and clinical performance of the nomogram. The feature importance of variables in the model is calculated based on the XGboost method. Restricted cubic splines (RCS) were used to explore the relationship between respiratory rate and hospital stay longer than 14 days.

### Statistical analysis

Continuous variables were represented by medians and quartiles, and differences between groups were determined using analysis of variance. Categorical variables were expressed as frequency and percentage values, and differences between groups were determined using chi-square tests. We used R software (4.2.0) for statistical analysis. The R packages used in this study include gtsummary (Table 1), dplyr, glmnet, foreign, ISwR, car, rms, regplot, pROC, nricens, forestmodel, and tidymodels.

**TABLE 1:** Baseline characteristics.

Variables	Total (*n* = 705)	Training cohort (*n* = 423)	Verification cohort (*n* = 282)	*p*-value
Gender (n,%)				0.296
Male	362 (51%)	224 (53%)	138 (49%)	
Female	343 (49%)	199 (47%)	144 (51%)	
Body temperature (°C)	36.70 (36.40, 36.90)	36.70 (36.50, 36.90)	36.60 (36.40, 36.90)	0.070
Heart Rate(beats/min)	128 (114, 138)	128 (112, 136)	130 (116, 138)	0.093
Respiration rate (beats/min)	30 (28, 34)	30 (26, 34)	30 (28, 34)	0.133
DBP (mmHg)	54 (46, 62)	54 (46, 64)	55 (45, 62)	0.248
SBP (mmHg)	98 (89, 106)	98 (89, 106)	98 (88, 107)	0.651
Spo2 (%)	98 (97, 99)	98 (97, 99)	98 (97, 99)	0.862
Adenosine deaminase	14.0 (11.7, 16.8)	14.0 (11.7, 16.8)	14.1 (11.8, 17.1)	0.743
Albumin	43.9 (41.7, 46.1)	43.8 (41.6, 46.0)	44.0 (41.7, 46.2)	0.512
ALT	18 (13, 27)	18 (13, 27)	18 (13, 26)	0.663
AST	41 (33, 57)	41 (32, 56)	41 (34, 58)	0.391
Urea	3.79 (2.66, 4.70)	3.80 (2.64, 4.71)	3.75 (2.71, 4.67)	0.657
Creatinine (umol/L)	43 (39, 48)	43 (39, 48)	44 (38, 47)	0.775
Tbil (umol/L)	7 (5, 10)	7 (5, 10)	6 (5, 10)	0.120
Globulin (g/L)	21.0 (18.0, 23.8)	21.3 (18.1, 23.8)	20.6 (17.7, 23.7)	0.235
Hemoglobin (g/dL)	119 (110, 126)	119 (110, 126)	118 (110, 126)	0.981
Lymphocyte count (×10^9/L)	5.06 (3.82, 6.62)	4.87 (3.63, 6.46)	5.22 (4.13, 6.80)	0.019
PLT (×10^9/L)	333 (271, 399)	331 (272, 394)	335 (267, 401)	0.650
RBC (×10^12/L)	4.42 (4.13, 4.67)	4.42 (4.14, 4.66)	4.42 (4.10, 4.69)	0.989
WBC (×10^9/L)	8.87 (7.30, 10.99)	8.78 (7.19, 10.98)	8.89 (7.65, 11.18)	0.173
Eosinophils (×10^9/L)	0.22 (0.13, 0.35)	0.23 (0.14, 0.36)	0.21 (0.13, 0.35)	0.460
D-dimer (mg/L)	0.21 (0.14, 0.39)	0.21 (0.15, 0.40)	0.21 (0.14, 0.38)	0.434
Fibrinogen (g/L)	1.97 (1.67, 2.32)	2.00 (1.65, 2.36)	1.96 (1.68, 2.23)	0.356
INR	0.98 (0.93, 1.03)	0.98 (0.93, 1.03)	0.98 (0.93, 1.03)	0.536
Bicarbonate (mmol/L)	22.10 (20.80, 23.30)	22.20 (20.80, 23.30)	22.00 (20.90, 23.30)	0.924
Na (mmol/L)	135 (133, 137)	135 (133, 137)	135 (133, 138)	0.801
BNP (pg/mL)	297 (106, 1,582)	283 (106, 1,492)	320 (108, 1,608)	0.858

## Results

**FIGURE 1:**
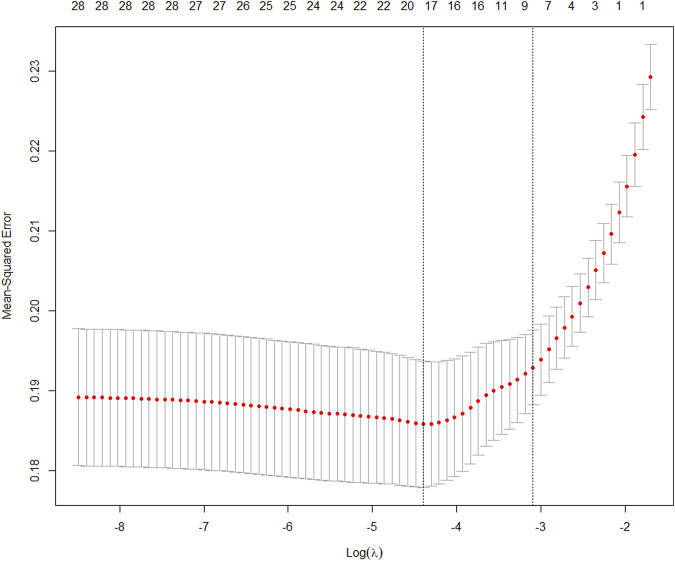
Different mean-squared error across the range of lambda.

**FIGURE 2:**
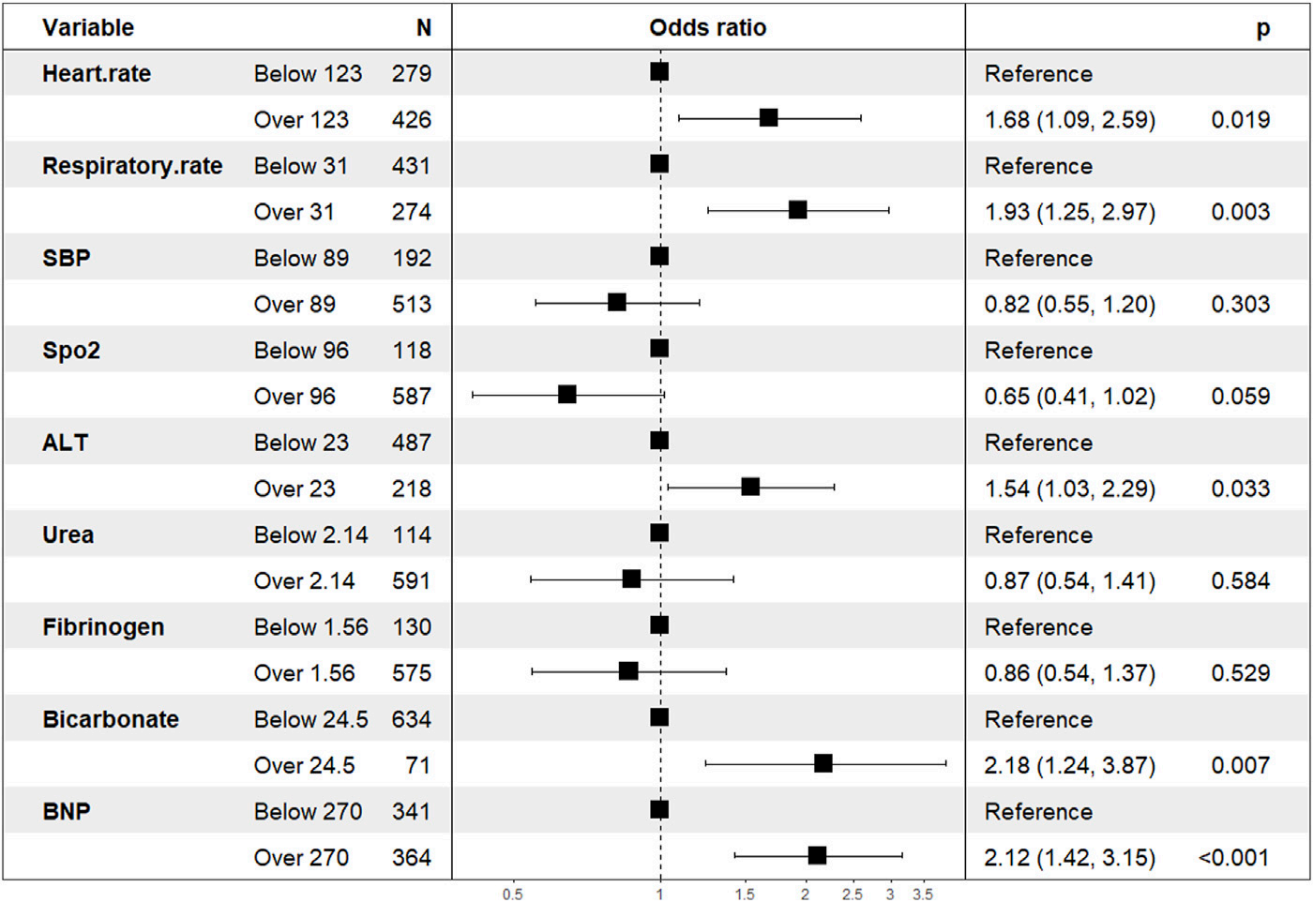
Variables that are ultimately included in the nomogram. Abbreviation: Spo2, blood oxygen saturation; SBP, systolic blood pressure; ALT, alanine aminotransferase; BNP: brain natriuretic peptide.

**FIGURE 3:**
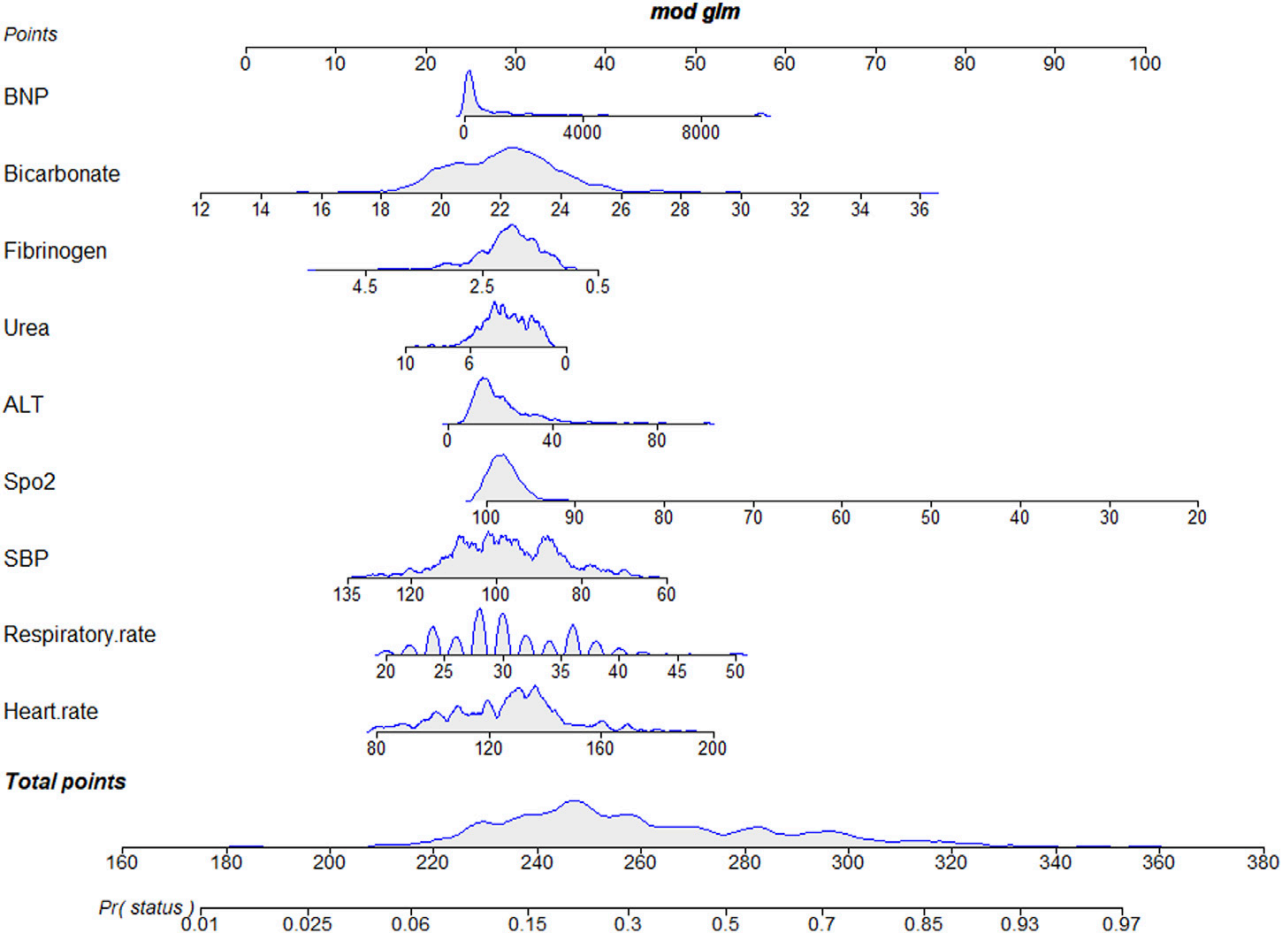
A nomogram for predicting the length of hospital stay in patients with VSD longer than 14 days.

**FIGURE 4:**
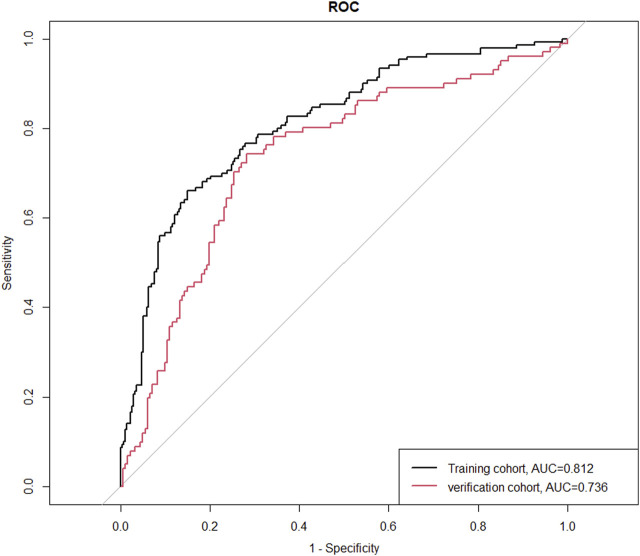
The ROC curves of the training cohort and the verification cohort. Abbreviations: ROC, receiver operating characteristic curve; AUC, area under the receiver operating characteristic curve.

**FIGURE 5:**
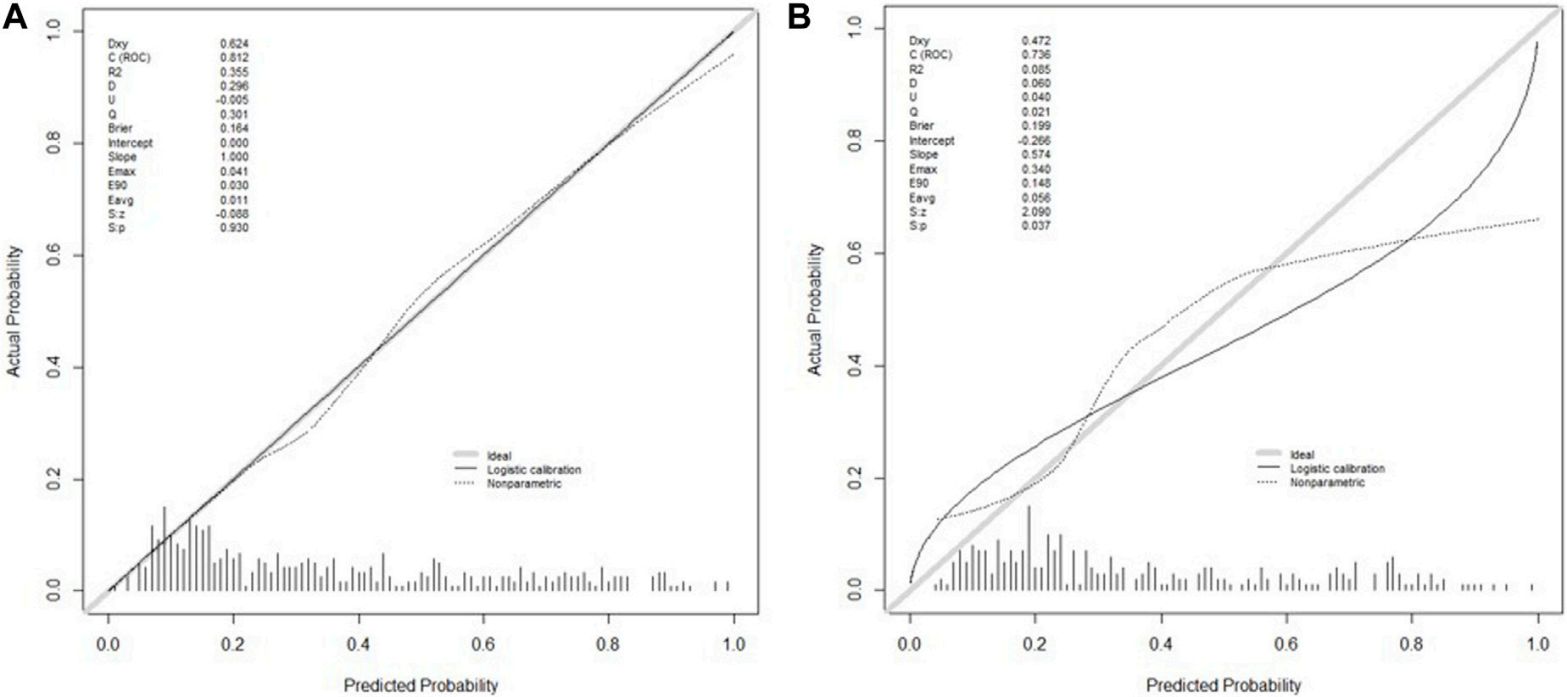
Calibration plots of the nomogram in the training cohort **(A)** and the validation cohort **(B)**.

**FIGURE 6:**
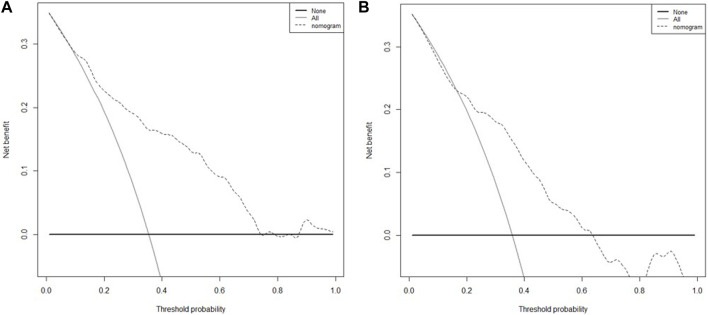
The DCA curve of patients with the nomogram, **(A)** training cohort; **(B)** validation cohort.

**FIGURE 7:**
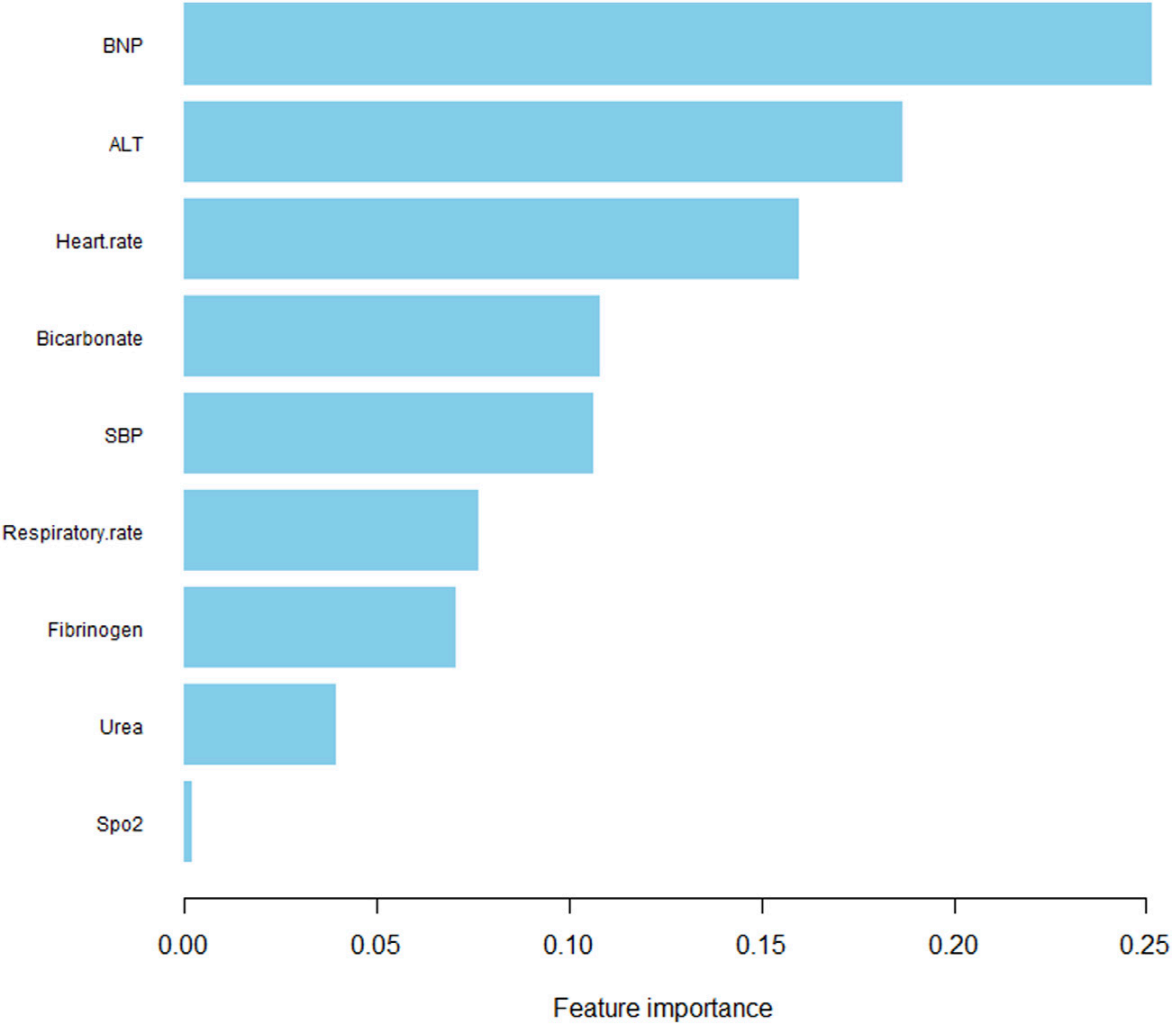
The importance of each variable.

We extracted data from the PIC database on 705 patients with a diagnosis of ventricular septal defect, 362 of whom were male. We randomly assigned 60% of patients to the training cohort and 40% to the validation cohort. Except for lymphocyte counts, baseline data for both cohorts were balanced (*p*-value >0.05). The baseline data of the two groups of patients are shown in Table 1. The VIF of all variables is less than 5, indicating that there is no multicollinearity among the variables. The VIF of the variable is shown in Supplementary Table S1; Figure 1 shows the different mean squared error within the range of log(lambda). Based on LASSO analysis, a total of 9 most likely non-zero coefficient characteristic variables were screened, including heart rate, respiratory rate, SBP, Spo2, urea, ALT, fibrinogen, bicarbonate, and BNP. The cutoff values for heart rate, respiratory rate, SBP, Spo2, urea, ALT, fibrinogen, bicarbonate and BNP were 123, 31, 89, 96, 23, 2.14, 1.56, 24.5, 270, respectively. The variables were incorporated into the logistic regression model to determine the Odds ratio, and the results were represented by a forest plot (Figure 2). The above 9 variables were incorporated into the logistic regression model, and a nomogram was established (Figure 3). The AUC of nomogram in the training cohort and validation cohort were 0.812 and 0.736, respectively, indicating that nomogram has good predictive performance (Figure 4). The calibration curve indicated that the nomogram had good consistency between the training and validation cohorts in predicting the length of hospital stay in patients with ventricular septal defect longer than 14 days (Figure 5). Figure 6 shows the DCA of the nomogram, showing a net benefits in both the training and validation cohorts. Figure 7 shows the importance of each variable, BNP is the most important variable in the nomogram.

## Discussion

Ventricular septal defect is the most common congenital heart disease caused by hypoplasia of the ventricular septum during the embryonic stage. In the early stage of ventricular septal defect, blood is shunted from the left to the right ventricle, which leads to an increase in the pulmonary blood circulation volume that causes pulmonary interstitial edema, which is prone to pulmonary infection.

Previous studies have shown that risk factors for unplanned readmission to pediatric intensive care units include age, disease type, disease severity, source of admission, unplanned initial admission, time of initial discharge, and respiratory support at discharge (Odetola et al., 2007; Linton et al., 2009; Bernard and Czaja, 2013; Czaja et al., 2013; Edwards et al., 2013; Mandell et al., 2015; Kaur et al., 2018). Most of these risk factors cannot be changed, but the length of hospital stay may be reduced with optimal treatment. This study identified risk factors for hospitalization longer than 14 days in children with ventricular septal defect, including heart rate, respiratory rate, systolic blood pressure, oxygen saturation, ALT, urea, fibrinogen, bicarbonate, and BNP. The risk of hospitalization for more than 14 days was increased when heart rate >123 beats/min, respiratory rate >31 beats/min, ALT >23 U/L, bicarbonate >24.5 mmol/L, and BNP >240 pg/mL.

When pulmonary infection or heart failure occurs in patients with ventricular septum, pulmonary interstitial edema and pulmonary diffusion dysfunction may occur, resulting in hypoxemia and decreased SpO_2_. The compensatory mechanism of the body deepens and accelerates the breathing rate, which often worsens the illness, so the risk of a hospital stay longer than 14 days increases. Respiratory frequency also plays an important role in monitoring infectious and respiratory diseases. Studies have found that respiratory rate is also an important prognostic factor in pulmonary infection and heart failure (Fischl et al., 1981; Aujesky et al., 2006; Bhatia et al., 2006). The respiratory rate has also been used as an important predictive value in assessing the severity and early stages of exacerbation of acute diseases (McCartan et al., 2021). A study of 705,928 patients found that the respiratory rate on admission had a U-shaped relationship with mortality, with a respiratory rate of 20 breaths/min at admission having the lowest mortality rate (Strauß et al., 2014). Studies have found that the degree of pneumonia infection is related to duration, and so early identification or early antibiotics use can significantly improve the survival rate (Weinstein et al., 1997; Balakrishnan et al., 2000; Rivers et al., 2001; Houck et al., 2004). Therefore, monitoring respiratory rate for early identification of lung infections may improve outcomes. RCS revealed a nonlinear relationship between respiratory rate and a stay longer than 14 days. A respiratory rate exceeding 30 breaths/min increases the risk of a patient being hospitalized for longer than 14 days.

Fibrinogen is a plasma protein coagulation factor synthesized and secreted by hepatocytes, which can participate in hemostasis and thrombosis, regulate coagulation and fibrosis, and prevent the spread of infection and inflammation (Davalos and Akassoglou, 2012). Studies have shown that elevated fibrinogen levels in patients with stable coronary artery disease increase the risk of myocardial infarction and long-term death (Mjelva et al., 2018; Zhang et al., 2020). Studies have shown that in patients with acute aortic dissection, lower fibrinogen levels are associated with an increased risk of in-hospital mortality (Liu et al., 2018). Our study had similar findings that fibrinogen >1.56 g/L was associated with a reduced risk of hospitalization longer than 14 days. In the initial stage of infection, an increase in fibrinogen may be a common finding. Possible mechanisms include activation of the coagulation system by the release of tissue factor when the vascular endothelium is disrupted, which consumes large amounts of fibrinogen and reduces its concentration in the blood. Lower fibrinogen levels imply overactivation of the coagulation system, which may prolong hospital stays and increase the risk of death. In addition, when disseminated intravascular coagulation (DIC) occurs, resulting in a large consumption of coagulation factors, including fibrinogen, it may also lead to poor prognosis.

We hope that clinicians can use the nomogram from this study to accurately identify high-risk groups in pediatric patients with VSD, and provide early interventions to reduce hospital stays, which can help reduce the risk of in-hospital complications and improve outcomes.

The limitations of this study were as follows: First, it had a single-center retrospective design, and its conclusions are not representative of all juvenile patients with ventricular septal defect. Second, we excluded some variables due to high rates of missing data. Due to database limitations, we did not include age, trisomy 21 syndrome, and Eisenmenger syndrome in the analysis. In future studies we hope to include troponin and other variables that can reflect myocardial injury, which may increase the accuracy of the findings.

## Conclusion

The nomogram we developed includes heart rate, respiratory rate, SBP, Spo2, ALT, Urea, fibrinogen, bicarbonate, BNP. The model has good predictive performance and clinical utility, and is convenient for clinicians to use. Therefore, the new model can help clinicians assess the risk of hospitalization for longer than 14 days in pediatric patients with VSD.

## Data Availability

Publicly available datasets were analyzed in this study. This data can be found here: http://pic.nbscn.org/.

## Abbreviations

ADA, Adenosine deaminase; ALT, Alanine aminotransferase; BNP, brain natriuretic peptide; DBP, Diastolic blood pressure; Hr, Heart rate; INR, International normalized ratio; Na, Serum sodium; PIC, Paediatric Intensive Care; PLT, Platelets; RCB, Red blood cells; Rr, Respiratory rate; SBP, Systolic blood pressure; Spo2, Blood oxygen saturation; T, Body temperature; Tbil, Total bilirubin; WBC, White blood cells.
