# Supplementary material for: Development and validation of a nomogram for predicting hospitalization longer than 14 days in pediatric patients with ventricular septal defect—a study based on the PIC database
Source: Front Physiol. 2023 Jul 4;14:1182719. doi: 10.3389/fphys.2023.1182719 (PMC10352838; doi:10.3389/fphys.2023.1182719)
Supplement: Supplementary file 1 [file Table1.docx]

| Variables | VIF |
| --- | --- |
| Body temperature | 1.062056 |
| Heart rate | 1.406645 |
| Respiration rate | 1.948518 |
| DBP | 1.704521 |
| SBP | 1.67325 |
| Spo2 | 1.103982 |
| ADA | 1.451471 |
| Albumin | 1.519244 |
| ALT | 1.885914 |
| urea | 1.52528 |
| Creatinine | 1.476973 |
| TBil | 1.565321 |
| Globulin | 1.951153 |
| Hemoglobin | 2.520975 |
| Lymphocytecount | 3.971477 |
| PLT | 1.27429 |
| RBC | 2.57909 |
| WBC | 4.335522 |
| Basophil | 1.196517 |
| Monocyte | 1.732915 |
| Eosinophils | 1.266423 |
| Ddimer | 1.147041 |
| Fibrinogen | 1.590621 |
| INR | 1.483139 |
| Bicarbonate | 1.156805 |
| Na | 1.122349 |
| BNP | 1.863944 |

Table S1. Variance inflation factor for the variable
